# Arsenic trioxide induces apoptosis and the formation of reactive oxygen species in rat glioma cells

**DOI:** 10.1186/s11658-018-0074-4

**Published:** 2018-03-27

**Authors:** Yuanyuan Sun, Chen Wang, Ligang Wang, Zhibo Dai, Kongbin Yang

**Affiliations:** 1 Nursing Support Center, First Affiliated Hospital, Harbin Medical University, Harbin, 150000 China; 2 Neurosurgery Department, First Affiliated Hospital, Harbin Medical University, Nangang District, Harbin, 150000 China

**Keywords:** Arsenic trioxide (As_2_O_3_), Reactive oxygen species (ROS), Glioma, Apoptosis

## Abstract

**Background:**

Arsenic trioxide (As_2_O_3_) has a dramatic therapeutic effect on acute promyelocytic leukemia (APL) patients. It can also cause apoptosis in various tumor cells. This study investigated whether As_2_O_3_ has an antitumor effect on glioma and explored the underlying mechanism.

**Results:**

MTT and trypan blue assays showed that As_2_O_3_ remarkably inhibited growth of C6 and 9 L glioma cells. Cell viability decreased in glioma cells to a greater extent than in normal glia cells. The annexin V-FITC/PI and Hoechest/PI staining assays revealed a significant increase in apoptosis that correlated with the duration of As_2_O_3_ treatment and occurred in glioma cells to a greater extent than in normal glial cells. As_2_O_3_ treatment induced reactive oxygen species (ROS) production in C6 and 9 L cells in a time-dependent manner. Cells pretreated with the antioxidant N-acetylcysteine (NAC) showed significantly lower As_2_O_3_-induced ROS generation. As_2_O_3_ significantly inhibited the expression of the anti-apoptotic gene Bcl-2, and upregulated the proapoptotic gene Bax in both C6 and 9 L glioma cells in a time-dependent manner.

**Conclusions:**

As_2_O_3_ can significantly inhibit the growth of glioma cells and it can induce cell apoptosis in a time- and concentration-dependent manner. ROS were found to be responsible for apoptosis in glioma cells induced by As_2_O_3_. These results suggest As_2_O_3_ is a promising agent for the treatment of glioma.

## Background

Despite commonly being known as a toxic metalloid, arsenic trioxide (As_2_O_3_) has applications in traditional medicine in China. As early as the 1970s, a research group at the First Affiliated Hospital of Harbin Medical University discovered that As_2_O_3_ can induce remissions in up to 70% of acute promyelocytic leukemia (APL) patients [1, 2]. The dramatic therapeutic effect of As_2_O_3_ on APL was achieved primarily through the induction of cell differentiation and apoptosis [2, 3]. At low concentrations, As_2_O_3_ promoted cell differentiation, while at concentrations above 0.5 μmol/l, it induced cell apoptosis [4, 5].

As_2_O_3_ induced apoptosis not only in NB4 cells (an APL cell line) but also in various other tumor cell lines [6, 7]. The underlying mechanism remained unclear, but inhibition of cell differentiation and growth and induction of apoptosis are speculated to be the general mechanisms for tumor treatment [8] and As_2_O_3_ action [9, 10]. Further research on As_2_O_3_ in APL showed that reactive oxygen species (ROS) play an important role in the induction of apoptosis, and that APL cells are sensitive to the intracellular ROS levels [11]. However, there is still some discussion about whether ROS are involved in As_2_O_3_ inhibition of the growth of tumor cells [11–14].

Due to the existence of the blood–brain barrier, it is hard for therapeutics drugs to affect glioma cells. New therapeutics are required to overcome this challenge. Although it is still unclear how As_2_O_3_ could cross the blood–brain barrier, several studies of As_2_O_3_ in glioma indicate that it is a potential therapeutic agent for this type of cancer [9, 15].

The effective concentrations of As_2_O_3_ applied in those studies were extremely high, ranging from 4.0 μM to 5.0 mM [16, 17]. High concentrations of As_2_O_3_ carry a major health risk. Side effects include mild gastrointestinal discomfort, transient elevation of liver enzymes, reversible neuropathy, hypokalemia, hyperglycemia and cardiac toxicity. Prolongation of the life quality has been detected in as many as 38% of patients treated with As_2_O_3_ [18, 19]. In this study, we investigated the anti-tumor effect of a low concentration range (0–8 μmol/l) of As_2_O_3_ in the glioma cell lines C6 and 9 L, assessed changes to non-tumor (glial) cells, and explored the underlying mechanism by studying ROS.

## Methods

### Cell culture

As_2_O_3_ was obtained from Yida. Stock solutions were prepared in phosphate buffered saline (PBS) to exclude any unknown influence from other solvents. Working solutions were diluted in RPMI-1640 medium (Gibco) and Dulbecco’s modified Eagle’s medium (DMEM; Gibco), supplemented with 10% heat-inactivated fetal calf serum (FCS).

Rat C6 and 9 L glioma cells were obtained from Harbin Medical Neurosurgical Institute and were respectively cultured in 10% RPMI-1640 medium and 10% DMEM, in both cases supplemented with 10% FCS. Primary glial cells were isolated from new suckling Wistar mice within 24 h of birth using the method of McCarthy and de Vellis [20]. The cell concentration was adjusted to 5 × 10^5^ cells/ml in 15% DMEM. The fourth generation (after about 20 days of culture) was used. The cells were maintained at 37 °C, 95% air and 5% CO_2_ in a humidified incubator (Heraeus).

### Determination of cell viability

To test cell viability, cell suspensions of 2 × 10^5^ cells/ml were mixed with 0.4% trypan blue. After 5–10 min, dye exclusion was examined for viable cells under a light microscope. The 3-(4,5-dimethylthiazol-2-yl)-2,5-diphenyltetrazolium bromide (MTT) bromide assay was also used to determine the number of viable cells after exposure to As_2_O_3_. 200 μl cell suspensions (4 × 10^4^ cells/ml) were seeded in 96-well plates. Serially diluted As_2_O_3_ was added at final concentrations of 0 (control), 0.5, 1.0, 3.0, 5.0, 6.0, 7.0 and 8.0 μmol/l. Each experiment was performed in quadruplicate and repeated at least three times. After 24, 48 and 72 h, the MTT products were quantified and the results were presented as the percentage of viable cells and normalized to the level of controls. The optimal concentration was determined as 5.0 μmol/l and used to treat the rat C6 and 9 L cells.

### Measurement of apoptosis

After cultured for 24, 48 and 72 h, cell apoptosis was assessed using propidium iodide (PI) and annexin-V conjugated to fluorescein isothiocyanate (FITC) according to the manufacturer’s instructions (BD Biosciences). Briefly, cells with or without As_2_O_3_ were incubated with FITC-conjugated annexin-V. Then, the cells were collected, washed and centrifuged at 200 g for 10 min. The cell pellet was gently resuspended in 200 μl PI and incubated in the dark for 30 min at room temperature. Apoptosis was then assessed using flow cytometry.

Cell apoptosis and necrosis were further examined by staining with Hoechst 33,342 (HOE) and PI, respectively. Cells were plated into 96-well plates and treated with 5.0 μmol/l As_2_O_3_ for 24, 48 and 72 h. Cells (5 × 10^6^ cells/ml) were incubated for 15 min at 37 °C with HOE (10 μg/ml in PBS), centrifuged, washed in PBS, and resuspended at density of 1 × 10^7^ cells/ml. PI (50 μg/ml in PBS) was added before observation. Cells were examined using a light microscope (Olympus) equipped with a fluorescent light source and a UV-2A filter cube with an excitation wavelength of 330–380 nm and a barrier filter of 420 nm. All experiments were repeated at least three times.

### Measurement of ROS levels

The generation of ROS was measured as previously described [21]. Briefly, cell suspensions (2 × 10^6^ cells/ml) were exposed to As_2_O_3_ at 5.0 μmol/l for 24, 48 and 72 h. To evaluate the major organelles that governed the ROS-mediated stress in glioma cells, C6 and 9 L cells were pretreated with 5 nM antioxidant N-acetylcysteine (NAC) for 2 h, and were exposed to As_2_O_3_ at 5.0 μmol/l for 24 h [22]. After exposure, cells were incubated in 10 μM of 2′,7′-dichlorofluorescein diacetate (DCFH-DA; Molecular Probes) at 37 °C for 30 min. The cells were harvested and washed with cold PBS three times. Then, ROS levels were determined through fluorescence-activated cell sorting.

### Measurement of apoptotic proteins

Levels of apoptosis-related proteins (Bcl-2, Bax and Fas) were analyzed using Western blot as previously described [23]. Briefly, cells were lysed at 4 °C via RIPA. Proteins were separated using 10% SDS-PAGE, transferred to nitrocellulose membranes and incubated with primary antibodies against Bcl-2, Bax, Fas and actin (1:100, Santa Cruz Biotechnology). Then, the membranes were incubated with horseradish peroxidase-conjugated secondary antibodies, and detected using an enhanced chemiluminescence (ECL) kit (Beyotime).

### Statistical analysis

All quantitative data measurements were performed in triplicate and the results are presented as means ± standard deviation. One-way analysis of variance (ANOVA) was performed. The post hoc tests were Dunnett’s tests. Probability values (p) less than 0.05 were considered statistically significant.

## Results

### As_2_O_3_ decreased cell viability in C6 and 9 L glioma cells

**Fig. 1:**
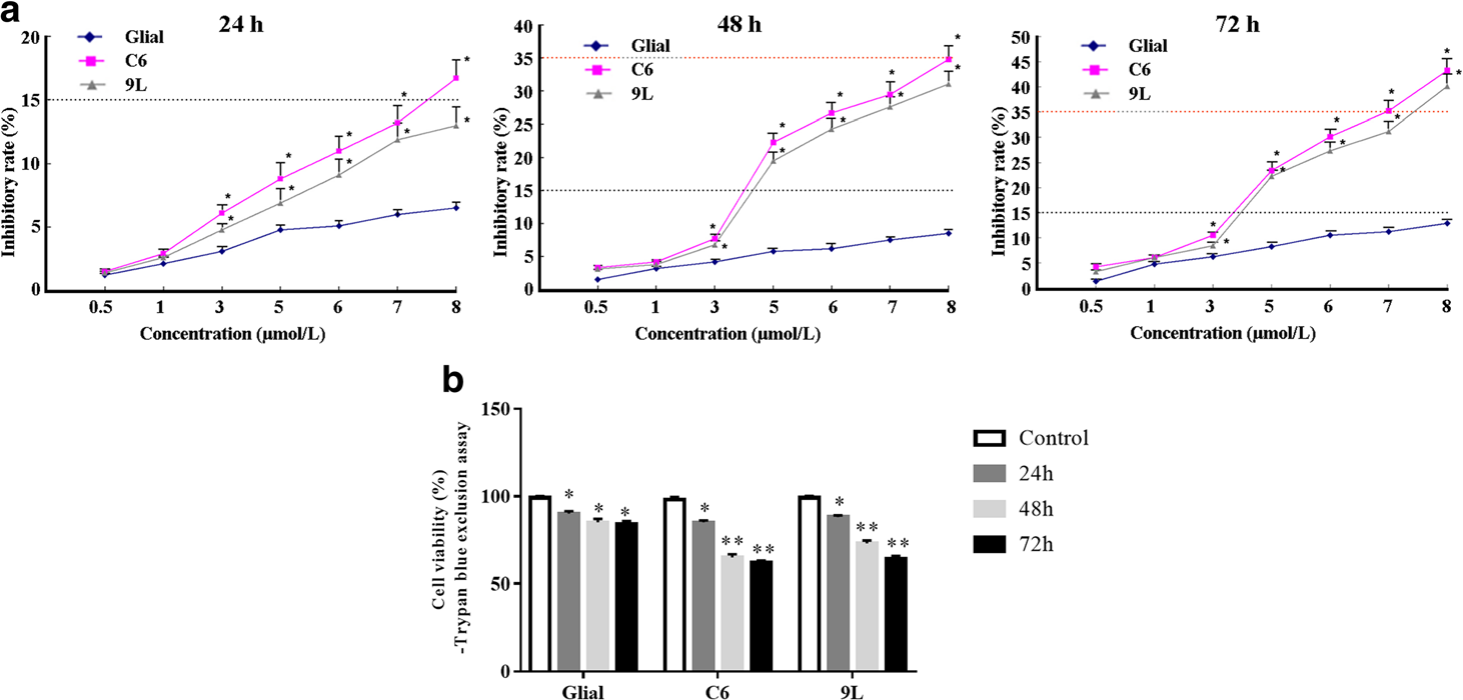
The inhibitory effects of As_2_O_3_ on the cell viability of C6 glioma cells, 9 L sarcoma cells and normal non-tumor (glial) cells (control). **a** The inhibitory rate was determined using the MTT assay 24, 48 and 72 h after exposure to As_2_O_3_ and it is expressed as % of control exposure at 24 h. **b** Cell viability was determined using the Trypan blue exclusion assay. *p < 0.05, **p < 0.01 vs. glial cells

The cytotoxicity of As_2_O_3_ in C6 and 9 L cells was assessed using the MTT and trypan blue assays. As_2_O_3_ was applied at 0.5, 1.0, 3.0, 5.0, 6.0, 7.0 and 8.0 μmol/l, and the inhibitory rates were determined after 24, 48 and 72 h (Fig. 1). The MTT assay showed that the As_2_O_3_-induced inhibitory rates for C6 and 9 L cells were dose and time dependent (Fig. 1a). The inhibitory effects of As_2_O_3_ on C6 and 9 L cells were significantly stronger than on normal glial cells. For example, the inhibition rate for normal glial cells exposed to 5.0 μmol/l As_2_O_3_ was less than 10% of that for glioma cells, suggesting that As_2_O_3_ inhibited the growth of glioma cells but not normal glial cells in range of 0–8 μmol/l. The calculated IC_50_ values for C6 and 9 L cells were respectively 5.0 and 5.6 μmol/l As_2_O_3_, so 5.0 μmol/l As_2_O_3_ was used in the following experiments.

The trypan blue assay showed that 5.0 μmol/l As_2_O_3_ significantly decreased cell viabilities in C6 and 9 L in a time-dependent manner (Fig. 1b). Although the cell viability in normal glial cells was also significantly decreased, the change was smaller than for glioma cells, suggesting a greater inhibitory role in glioma than in glial cells.

### As_2_O_3_ induced apoptosis in C6 and 9 L glioma cells

**Fig. 2:**
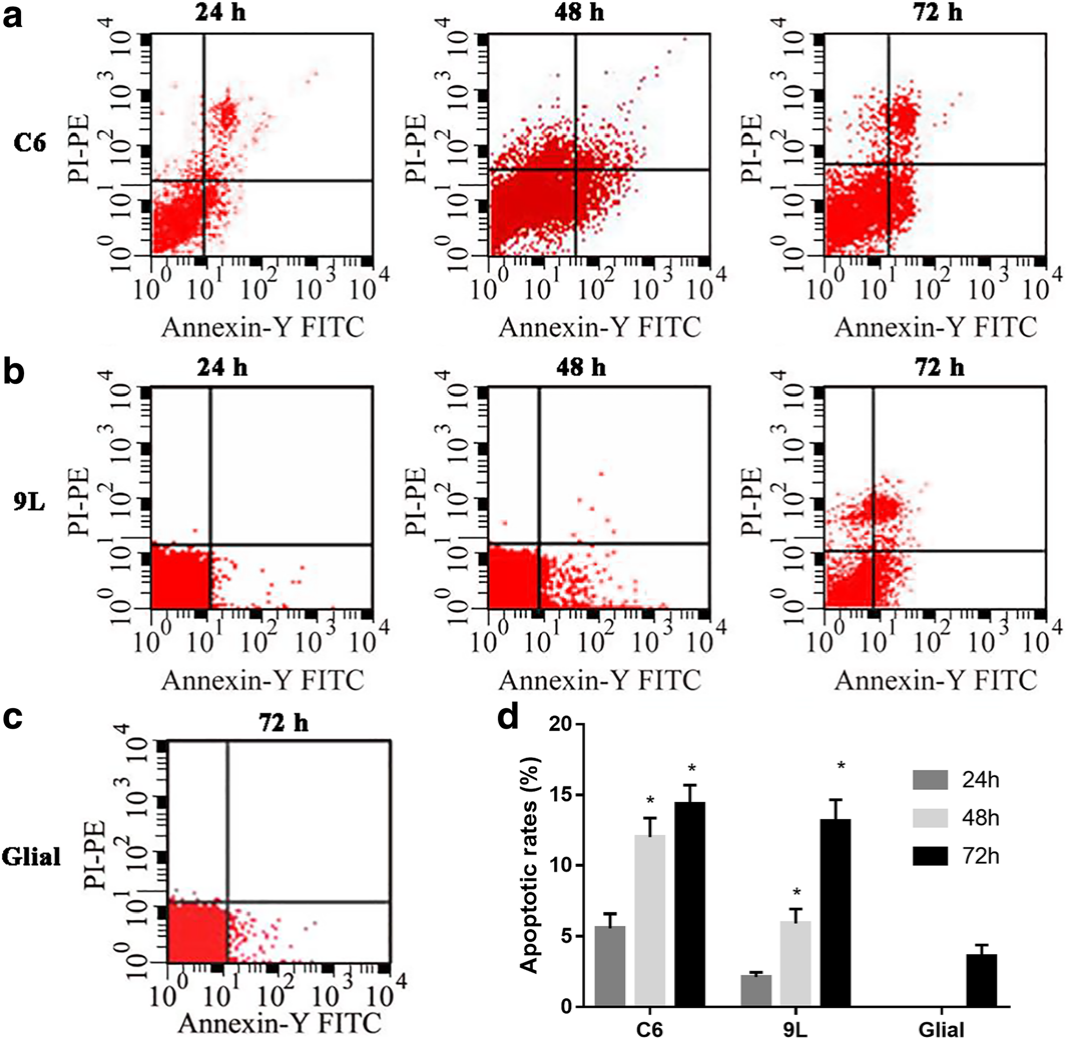
The annexin V-FITC/PI assay for apoptotic and necrotic cells. Cells were stained with annexin V-FITC and PI, and analyzed using a FACSScan flow cytometer. **a** C6 cells were treated with 5 μmol/l As_2_O_3_ for 24, 48 and 72 h. The apoptotic rate was 5.57, 12.01 and 14.35%, respectively. **b** 9 L cells were treated with 5 μmol/l As_2_O_3_ for 24, 48 and 72 h. The apoptotic rate was 2.12, 5.92 and 13.13%, respectively. **c** Glial cells were treated with 5 μmol/l As_2_O_3_ for 72 h. The apoptotic rate was 3.59%. **d** The quantification data. *p < 0.05 vs. glial cells

An annexin V-FITC/PI assay was used to assess cell apoptosis of glioma cells after exposure to 5 μM As_2_O_3_. The numbers of early (annexin V+/PI–) and late (annexin V+/PI+) apoptotic cells were calculated. In both C6 and 9 L glioma cells, apoptosis (seen as both early and late apoptotic cells) was significantly induced by 5 μM As_2_O_3_ in a time-dependent manner (Fig. 2a and b). The maximal percentages of apoptotic cells in both cell lines were reached at 72 h (14.35% of C6 cells, and 13.13% of 9 L cells; Fig. 2). However, the apoptotic rate for glial cells was only 3.59% (Fig. 2c and d). For cells without As_2_O_3_ treatment, the apoptosis rate was close to 0 (data not shown).

**Fig. 3:**
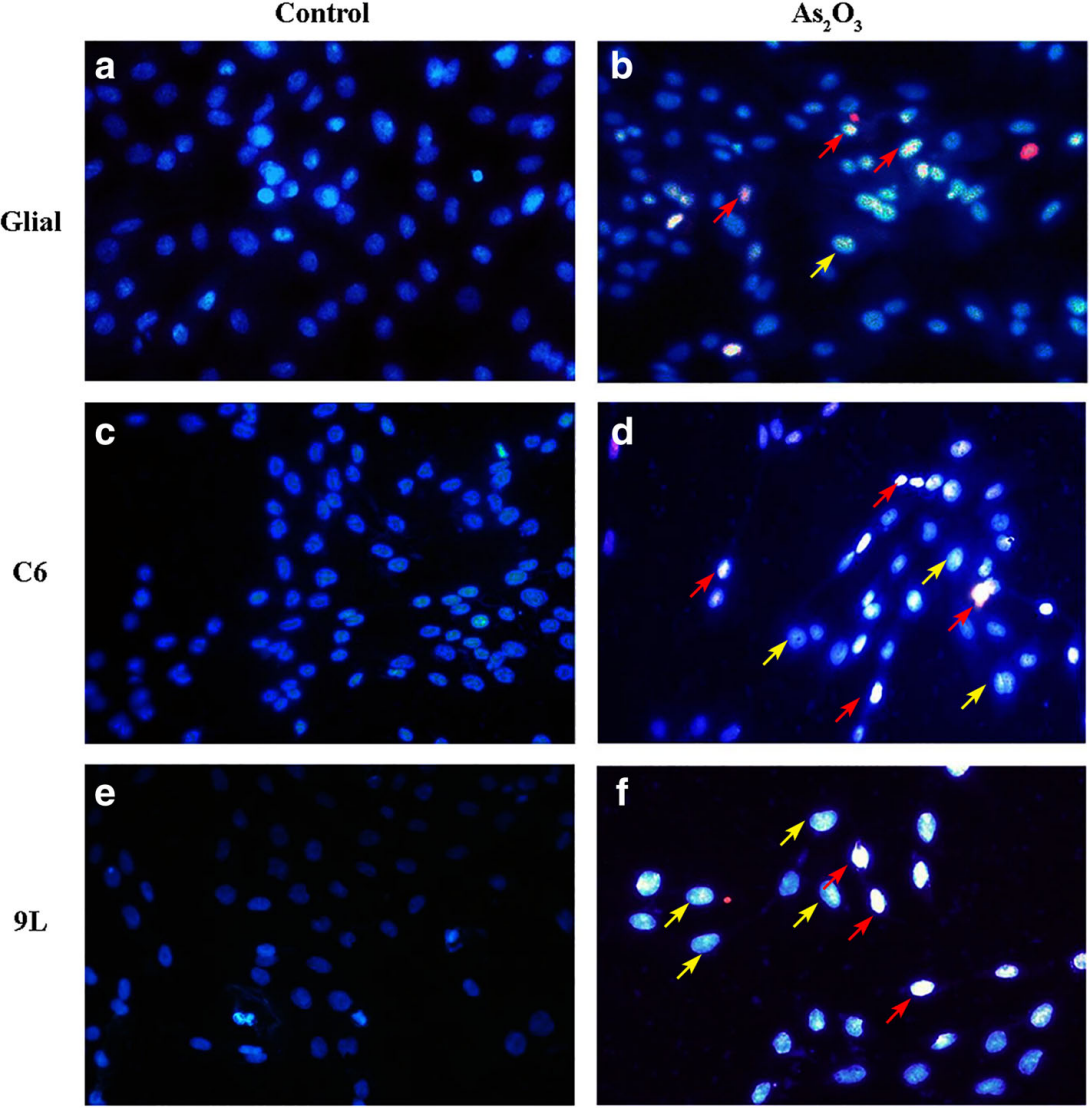
HOE/PI staining assay in glial (**a, b**), C6 glioma (**c, d**) and 9 L sarcoma (**e, f**) cells before (**a, c, e**) or after (**b, d, f**) treatment with 5 μmol/l As_2_O_3_ for 72 h. Magnification: 60×. Red arrows indicate necrosis. Yellow arrows indicate apoptosis. As_2_O_3_ might change the cell morphology of 9 L cells

The apoptosis in glial and glioma cells after exposure to 5 μM of As_2_O_3_ for 72 h was further confirmed with HOE/PI double staining (Fig. 3). Cell uptake of PI indicated necrosis. Cells with clear nuclear condensation but no PI uptake indicated apoptosis. After exposure to As_2_O_3_ for 72 h, C6 and 9 L cells showed increases in both necrosis and apoptosis. The level of apoptosis and necrosis induced by As_2_O_3_ was higher in C6 and 9 L cells than in glial cells.

### Production of ROS in C6 and 9 L glioma cells exposed to As_2_O_3_

**Fig. 4:**
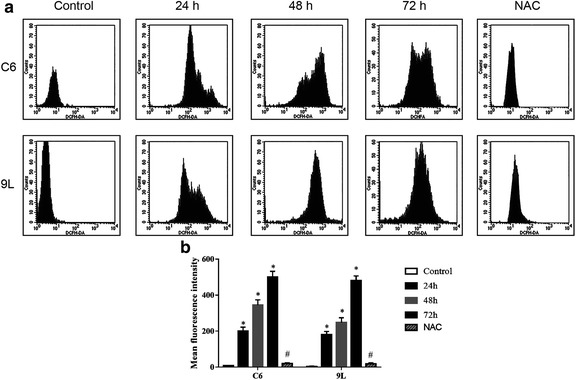
Effects of As_2_O_3_ on ROS generation in C6 and 9 L cells as measured using DCFH-DA. **a** C6 cells without exposure to 5 μmol/l As_2_O_3_ showed a mean fluorescence intensity of 7.58, while those with exposure for 24, 48 and 72 h showed 200.37, 344.80 and 501.74, respectively. 9 L cells without exposure to 5 μmol/l As_2_O_3_ showed a mean fluorescence intensity of 3.01, while those with exposure for 24, 48 and 72 h showed 180.27, 248.32 and 485.90, respectively. **b** Quantification data of ROS. Cells pretreated with NAC significantly inhibited the 24 h As_2_O_3_ increase in ROS levels. *p < 0.05 vs. control, #*p* < 0.05 vs. 24 h

The extent of cellular oxidative stress in living cells was estimated by monitoring ROS generation using the fluorescent dye DCFH-DA (Fig. 4). The mean fluorescence intensity in C6 cells was 7.58, 200.37, 344.80 and 501.74 at 0, 24, 48 and 72 h, respectively. The mean fluorescence intensity in 9 L cells was 3.01, 180.27, 248.32 and 485.90 at 0, 24, 48 and 72 h, respectively. Thus, the level of ROS level positively correlates with DCF intensity. In both C6 (Fig. 4a and b) and 9 L (Fig. 4b and c) cells, intracellular ROS increased significantly with increasing incubation time with 5 μmol/l As_2_O_3_ (*p* < 0.01). Cells pretreated with NAC significantly inhibited the increase in ROS in response to 24 h exposure to As_2_O_3_ (Fig. 4a and b).

### Effects of As_2_O_3_ on the expression of apoptotic proteins Bcl-2, Bax and Fas

**Fig. 5:**
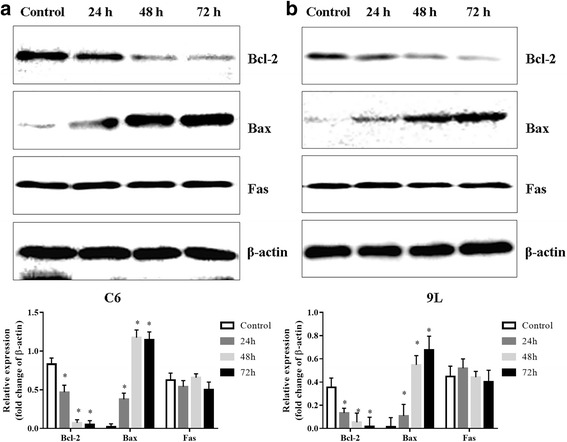
Western blot of Bcl-2, Bax and Fas in C6 glioma and 9 L sarcoma cells with or without treatment with As_2_O_3_ (5 μmol/l) for 24, 48 and 72 h. Bcl-2 expression reduced in a time dependent manner, while Bax expression increased in a time dependent manner. **a** Blots and quantification data for C6 cells. **b** Blots and quantification data for 9 L cells. *p < 0.05 vs. control

To validate the apoptosis process, the expression levels of apoptosis markers, including Bcl-2, Bax and Fas, were examined in C6 and 9 L glioma cells using western blotting. As_2_O_3_ significantly inhibited expression of the anti-apoptotic gene Bcl-2 and upregulated the pro-apoptotic gene Bax in both C6 and 9 L glioma cells in a time-dependent manner (Fig. 5a and b). The expression of Fas did not significantly change after exposure to As_2_O_3_ (Fig. 5a and b).

## Discussion

Because of its ability to induce apoptosis in various malignant tumor cells, As_2_O_3_ has potential as a treatment agent for malignant tumors [24, 25]. Gliomas are highly aggressive tumors that respond poorly to existing clinical therapeutic agents. In previous studies, it was shown that As_2_O_3_ treatment could inhibit cell growth of glioma cells, but the studies did not yield guidance on the effective doses [26–28].

Here, we investigated the effective doses of As_2_O_3_ using rat glioma cells and comparing them with non-tumor glial cells. Our results showed that As_2_O_3_ inhibited the growth of glioma cells in time- and concentration-dependent manners, and that 5.0 μmol/l As_2_O_3_ is the optimum concentration for inhibiting cell viability in both C6 and 9 L glioma cells. The inhibitory rate for non-tumor cells was less than 10% of that for the glioma cells, indicating that As_2_O_3_ is a promising drug. Due to the exist of the blood–brain barrier, it remains unclear how the 5 μmol/l concentration can be obtained in human blood such that it would be useful for treating glioma cells. Further studies using in vivo animal models are needed.

Both the HOE/PI and annexin-V/PI assays showed that 5.0 μM As_2_O_3_ induced apoptosis. However, the mechanism of apoptosis in solid tumor cells is far from clear. In glioma cells treated with As_2_O_3_, one of the most likely mechanisms for triggering an antitumor effect is the induction of ROS [29, 30]. Like other heavy metals, including iron, copper, chromium, cadmium, lead and mercury, arsenic affects cells by causing oxidative damage, primarily through disruption of the endogenous cellular antioxidant–redox balance [29, 30]. Cysteine thiol is the functional site for most redox proteins. Arsenic can directly bind to this site and destroy protein function, thereby affecting ROS production and clearance [29, 30]. Cell viability, ROS levels, apoptosis and autophagy in human glioblastoma cell line have been shown to be regulated by As_2_O_3_ [31, 32] and/or As_2_O_3_ in combination with other agents [33]. As_2_O_3_ induces ROS production and apoptosis in glioma cells through the upregulation of the mitoferrin-2 gene [34]. Consistently with the results of those studies, we also found that intracellular ROS levels increased significantly after As_2_O_3_ treatment.

The brain appears to be especially sensitive to ROS stress when compared to other organs. Although comprising only 2% of human body weight, the human brain consumes up to 20% of the oxygen supply. Such a high level of oxygen consumption indicates that large quantities of ROS are generated during oxidative phosphorylation in brain tissue. In addition, iron content has been shown to increase brain sites in which ROS production may be greater [35]. Tumor cells are vulnerable to ROS stress. Thus, therapeutic approaches directed at ROS intervention may have an antitumor effect, and As_2_O_3_ is a promising antitumor reagent for gliomas.

As_2_O_3_ downregulated the expression of Bcl-2, an anti-apoptotic protein, and upregulated the expression of Bax, a pro-apoptotic protein, thus shifting the Bax/Bcl-2 ratio in favor of apoptosis. Fas protein expression remained unchanged. These findings indicate that Bcl-2 and Bax play an important role in As_2_O_3_-induced apoptosis in C6 and 9 L glioma cells.

Our results hinted at the possible involvement of mitochondrial dysfunction in As_2_O_3_-induced apoptosis. The Bcl-2 family of proteins appear to control cell death by regulating mitochondrial physiology [36]. A change in the mitochondrial electrochemical potential results in the release of apoptotic proteins, such as cytochrome c, Smac/DIABLO, pro-caspases 2, 3 and 9, and apoptosis-inducing factor.

Under physiological and pathophysiological conditions, ROS contributes to trigger and mediate apoptosis [37]. The mitochondria are highly susceptible to oxidative damage, and Bcl-2 exerts its anti-apoptotic function by reducing intracellular ROS. As_2_O_3_ downregulated Bcl-2 and rendered C6 and 9 L glioma cells vulnerable to apoptotic cell death. In cells pretreated with NAC, As_2_O_3_-induced apoptosis was inhibited, suggesting that a mitochondrial death pathway plays an important role in As_2_O_3_-induced apoptosis.

## Conclusion

As_2_O_3_ strongly inhibits cell viability and induces apoptosis of rat C6 and 9 L glioma cells in vitro when used at an optimal concentration of 5 μmol/l. This action is related to the induction of ROS generation. Moreover, As_2_O_3_ showed lower cytotoxicity to normal glial cells than glioma cells, indicating that As_2_O_3_ may be a potentially potent chemotherapeutic agent for treating brain tumors.

## Abbreviations

APL: Promyelocytic leukemia
As_2_O_3_: Arsenic trioxide
DMEM: Dulbecco’s modified Eagle’s medium
ECL: Enhanced chemiluminescence
FCS: Fetal calf serum
FITC: Fluorescein isothiocyanate
MTT: 3-(4,5-dimethylthiazol-2-yl)-2,5-diphenyltetrazolium bromide
PBS: Phosphate buffered saline
PI: Propidium iodide
ROS: Reaction oxygen species
